# Association of CD8^+ ^T lymphocyte repertoire spreading with the severity of DRESS syndrome

**DOI:** 10.1038/srep09913

**Published:** 2015-04-23

**Authors:** Jun Niu, Qingzhu Jia, Qingshan Ni, Yi Yang, Gang Chen, Xichuan Yang, Zhifang Zhai, Haili Yu, Peng Guan, Regina Lin, Zhiqiang Song, Qi-Jing Li, Fei Hao, Hua Zhong, Ying Wan

**Affiliations:** 1 Department of Dermatology, Southwest Hospital, Third Military Medical University, Chongqing, China; 2 Biomedical Analysis Center, Third Military Medical University, Chongqing, China; 3 Chongqing Key Laboratory of Cytomics, Chongqing, China; 4 Department of Immunology, Duke University Medical Center, Durham, North Carolina, USA

## Abstract

T-cell receptor (TCR)-mediated cross-recognition is a major mechanism in the pathogenesis of drug reaction with eosinophilia and systemic symptoms (DRESS) syndrome. However, the characteristics of the TCR repertoire and the clinical significance of repertoire reformation throughout the course of DRESS are unknown. Here, we isolated CD4^+^ and CD8^+^ T-cells from peripheral blood of 8 DRESS patients at 10-day intervals and, sequenced CDR3-regions of the TCRB chain by high-throughput sequencing to analyze the dynamic reformation in the T-cell repertoire hierarchy. Compared with healthy donors, T-cell expanded in peripheral repertoires from DRESS patient. The extent of fluctuation of dominant CD8^+^ T-cell clones, but not of CD4^+^ counterparts, correlated positively with the clinical severity and helped classify the enrolled subjects into “fluctuant” and “flat” repertoire groups. The anti-herpesvirus response, which was measured using anti-EBV/HHV antibodies, and the proportion of the homologous CD8^+^ EBV-specific clonotypes, in the “fluctuant” group was substantial higher than that in the “flat” group. Furthermore, autoimmune sequelae were observed in a cured “fluctuant” patient. Collectively, the clinical relevance of the fluctuant CD8^+^ T-cell repertoires supports the notion that herpes virus-mediated continuously *de novo* priming of newly pathogenic CD8^+^ T-cell clones is an alternate mechanism responsible for the pathogenicity of DRESS.

Drug reaction with eosinophilia and systemic symptoms (DRESS) syndrome is a rare but life-threatening syndrome^1^, which was characterized by a long latency period and a prolonged disease course with frequent relapses despite the discontinuation of the culprit drug. Unique observations in DRESS patients include a cascade of viral reactivation (EBV, HHV-6, HHV-7, and CMV) and the development of autoimmune sequelae^2^. These clinical features implicate an underlying immune-mediated pathogenic mechanism in the process of DRESS.

The presence of allergen-specific T-lymphocytes is a universal clinical observation in most severe drug-allergy reactions. Blistering reactions are considered to be correlated with cutaneous infiltration of cytotoxic CD8^+^ T-cells^3^, whereas exanthemas seems to be mediated largely by drug-specific CD4^+^ T-cells^4^. Furthermore, cross-reactive injury on multiple organs by misguiding EBV-driven CD8^+^ T-cells have also been identified in DRESS^5^. On the other hand, immunosuppression is also frequently observed in early stage of DRESS syndrome. The marked over-representation of regulatory T-cells during the acute stage has been considered to explain the sequential reactivation of antiviral T-cells. Although accumulating evidence give static view into the pathogenic role of CD4^+^/CD8^+^ allergenic T-cell clones, the precise pathogenic mechanisms underlying the dynamic T-cell repertoire change throughout the prolonged disease remain poorly understood.

T-cell-mediated antigen-recognition critically depends on a highly diversified T-cell receptor (TCR) repertoire. The complementarity-determining region 3 (CDR3) region within the TCR is the most variable part of the molecule and determines the specificity and strength of binding of the interaction, thereby contributing to the diversity of the repertoire and allowing the receptor to recognize a variety of antigens^6^. In more recent studies, large-scale analyses of the CDR3 region of TCR using high-throughput sequencing have provided a detailed snapshot of the adaptive immune response^7^. In this study, we longitudinally characterized the CD4^+^/CD8^+^ T-cell repertoires in DRESS from diagnosis to clinical remission by using repertoire sequencing. In an attempt to improve personalized immunotherapy and early interventions, we analyzed (1) the expansion and signature of the peripheral repertoire of distinct T-cell compartments, (2) the dynamic reformation of the T-cell repertoire, and (3) the association between clinical features and T-cell repertoire reformation.

## Results

### Enrichment of expanded clones in DRESS patients

Principal component analysis indicated that the overall usage of the *TRBV/J* segment was highly dependent on the individual, without overt general bias in either DRESS patients or healthy donors (Supplementary Figure S2). These data suggested that an aberrant *TRBV/J* usage pattern is not a valid biomarker for diagnosis of DRESS. To further evaluate whether oligoclonal expansion of T-cell repertoire was evident in DRESS repertoire, we compared the proportion of highly expanded clones (HECs, defined as clonotypes having an abundance beyond a cutoff value in their repertoires) in peripheral blood between patients (sampling at diagnosis, without T-cell subset sorting) and healthy donors. To determine the “highly expanded” threshold, a gradient cutoff (ranging from 0.1% to 10%, at intervals of 0.1%) was introduced to avoid artificial bias. The proportion of expanded clones tended to show a clear increase, in DRESS patients, in contrast to those in healthy donors, demonstrating a considerable oligoclonal expansion in the DRESS circulatory repertoire, especially at higher HEC cutoff settings (Supplementary Figure S3A). To confirm these findings clearly, we performed statistical testing again in intervals of 1% (Supplementary FigureS3B, C) and identified consistent observations. Furthermore, we observed higher oligo-clonal expansion for DRESS (Supplementary Figure S4) when calculating the clonality of each T-cell repertoire^8^. These results suggested that the dominant clonotypes are substantially elevated in the patients’ repertoire relative to that in healthy donors, which strongly implicated the occurrence of oligoclonal proliferation events in DRESS.

### “Flat” and “fluctuant” dynamic pattern of CD8^+^ repertoires

**Figure 1:**
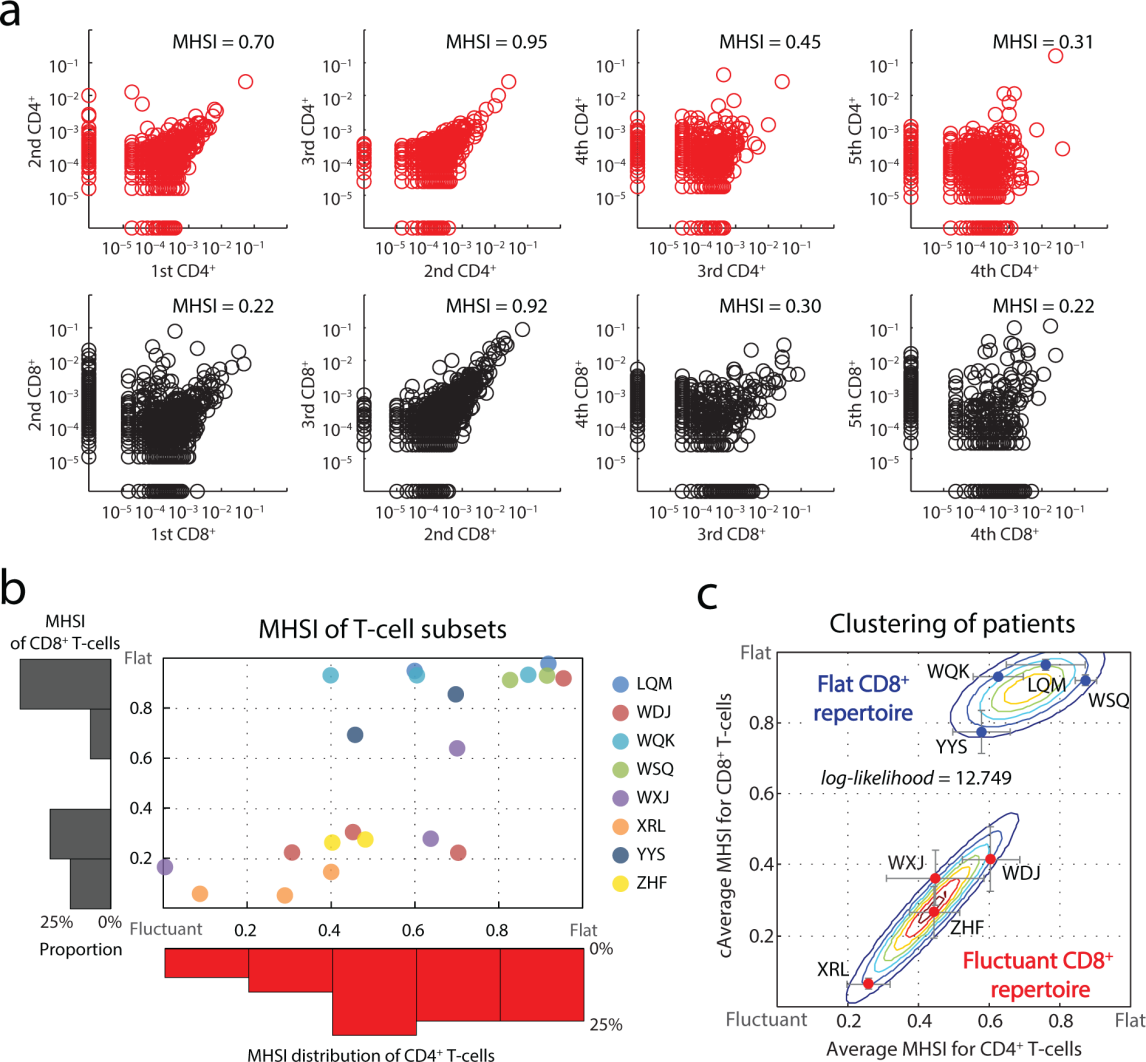
Dynamic repertoire composition of T-cell subsets. **(a)** Longitudinal repertoire reformation in 1 patient. Distribution of the CDR3 AA clonal abundance of CD4^+^ (upper panels, red) and CD8^+^ (lower panels, black) repertoires obtained at different sampling times from a single patient. Dots on the axis indicate the clonotypes detected within a single repertoire from 2 adjacent sampling points. The Morisita–Horn similarity index (MHSI) is shown. **(b)** Overall repertoire reformation for all patients. Each dot represents CD4^+^/CD8^+^ MHSI for each “disease interval” for each patient. The marginal histogram shows the distribution of the repertoire MHSI for CD4^+^ (horizontal, red) and CD8^+^ (vertical, black) T-cells. **(c)** Incorporated MHSI values and categorization for each patient. Dots represent the average CD4^+^/CD8^+^ repertoires MHSI from individuals. Error bar, STD. The expectation maximization algorithm was used to perform the clustering under Gaussian mixture models. The contour shows the estimated probability density for the two groups. The log-likelihood is shown.

To quantify repertoire dynamics over disease, we calculated the Morisita-Horn similarity index (MHSI, measuring the similarity of two repertoires, or, the reproducibility of single repertoire at different sampling)^9^ between every adjacent sampling across the duration of disease for each patient to indicate the extent of “repertoire reformation” in certain disease interval (Figure 1A and Supplementary Figure S5). Control-sequencing also demonstrate the reproducibility of our sequencing strategy, and, the stability of peripheral repertoire without immune-challenge (Supplementary Figure S6). All the MHSIs of patient’s CD4^+^/CD8^+^ subsets were represented as a scatter plot with a marginal histogram (Figure 1B). The pattern for the CD8^+^ T-cell repertoire tended toward a polarized distribution, being either persistently flat or markedly fluctuant. In contrast, CD4^+^ T-cells showed a uniform distribution without any potential bias (Figure 1C). Furthermore, clustering based on the average MHSIs of CD4^+^/CD8^+^ repertoire in all intervals for each patient demonstrated that the reformation intensities of the CD8^+^ repertoire hierarchy reliably categorized the DRESS patients into “flat” and “fluctuant” groups (Figure 1D).

**Figure 2:**
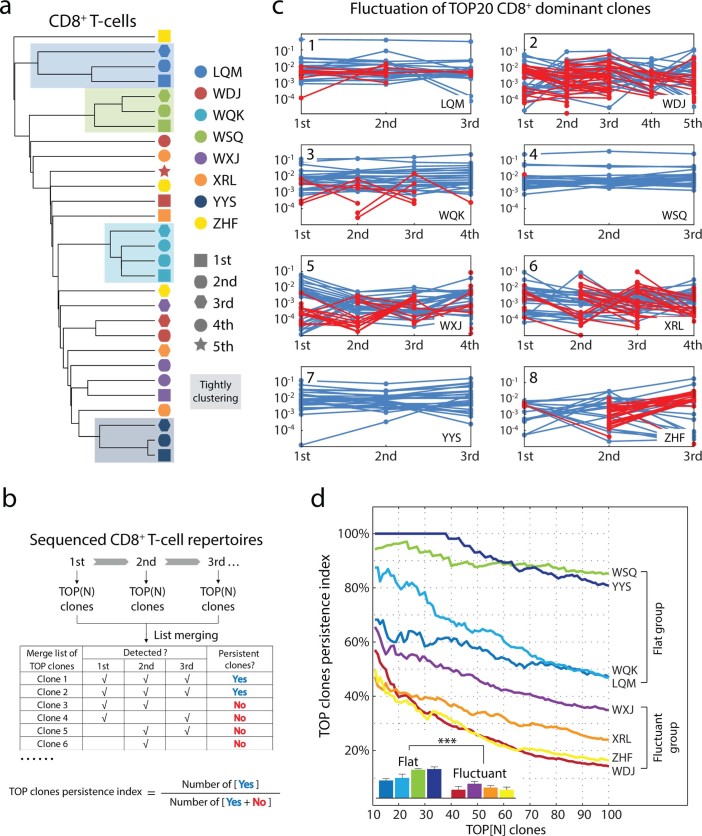
Clustering of fractioned blood samples. **(a)** Unsupervised clustering of CD8^+^ CDR3 AA clones across the sampling period. Light-colored backgrounds, individuals with tightly clustered samples. **(b)** Workflow for evaluating the “TOP clones persistence index (TCPI)” for each patient. Briefly, a defined number of most abundant clones was extracted from each sampling and was merged into a “TOP clones” list. “TOP clone” was marked as “Persistent” if the clonotype was found in every sampling across disease. The TCPI was defined as the ratio of “Persistent TOP clone” to merged “TOP clone”. Higher TCPI, flat repertoire; lower TCPI, fluctuant repertoire. **(c)** Repertoire fluctuation for TOP 20 dominant CD8^+ ^clones. “Persistent clones”, blue line; newly dominant clones, red line. **(d)** Fluctuation of CD8^+^ dominant CDR3 AA clones. N was assigned from 10 to 100. TCPI (as a function of N) were plotted against their N definition for each patient. Inset, grouped bars show the average index of each curve. Error bars, STD.

Given that the reproducibility of highly abundant T-cell clones is the dominant parameter in MHSI formulating, it is reasonable that the dichotomous aspect of the CD8^+^T-cellrepertoire hierarchy mainly reflects the disparate abilities to maintain the stability of dominant clones in the repertoires. To test this hypothesis, the compositions of CDR3 AA clonotypes in repertoires were clustered to illustrate the organization of these longitudinal samples (Figure 2A). The “flat” patients exhibited unique signatures (clustered closely) in binary tree, suggesting that these repertoires harboring coherent dominant clones throughout the overall disease. Conversely, the “fluctuant” patients did not exhibit such close organizations, but rather were arranged loosely in the dendrogram. These findings supported our previous analysis and suggested that the loss-of-reproducibility of dominant clones may make the main contribution to greater reformation in the CD8^+^ repertoire hierarchy.

To support this hypothesis, the persistence of the dominant CD8^+^ T-cell clones was directly examined using a “TOP clones persistence index (TCPI)” method (Figure 2B) to minimize the influence of sequencing error (Supplementary Figure S7). Consistently, the curves of the TCPI were significantly different between the “flat” and “fluctuant” patients (Figure 2C, D; and detail in Supplementary Table 4). The 4 “flat” patients harbored many more persistent dominant CD8^+^ clones than did the 4 “fluctuant” patients in the whole analysis range. Taken together, dynamic analysis of these repertoire datasets demonstrated that the extent of fluctuation of dominant CD8^+^ T-cells, but not of CD4^+^ T-cells divided the enrolled DRESS patients into “fluctuant” and “flat” groups.

### Clinical relevance of the fluctuation in CD8+ T-cells repertoire hierarchy

**Figure 3:**
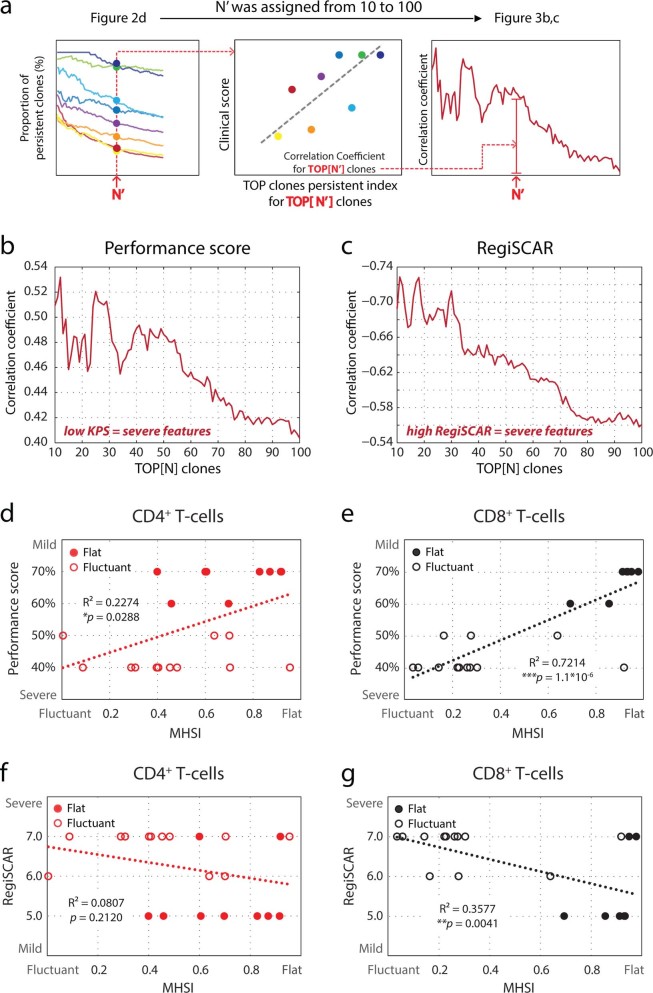
Clinical relevance of fluctuations in T-cell repertoires. **(a)** Workflow for investigating the clinical relevance of dominant clone substitution in **(b, c)**. For a given N’, the Pearson correlation coefficient was calculated for both Karnofsky performance scores (KPS) and RegiSCAR score, and was plotted as a function of N’. N’ was assigned from 10 to 100 most abundant CD8^+^ clones. **(b, c)** Correlation between fluctuation of dominant clones and clinical severity. **(d, e)** Correlations between KPS and fluctuations in each disease interval of CD4^+^/CD8^+^ repertoires. **(f, g)** Correlation between RegiSCAR score and fluctuations in each disease interval of CD4^+^/CD8^+^ repertoires. All clinical scoring was performed at the time of diagnosis and prior to steroid treatment. Repertoire fluctuation in each disease interval was measured by MHSI of 2 adjacent sampling points.

To assess the clinical relevance of the fluctuation in the CD8^+^ T-cell repertoire hierarchy, the association between the TCPI and clinical severity was firstly established (Figure 3A). By this analysis, we identified a positive correlation between fluctuation of dominant CD8^+^ clones (represented by lower TCPI) and severity of disease (Figure 3B, C). Furthermore, the association between MHSI (another measure of fluctuant intensity) and severe clinical features for CD8^+^ repertoires also confirmed these observations (Figure 3E, G). Additionally, this fluctuation-severity association was much more less to insignificant level for CD4^+^ T-cells (Figure 3D, F).

**Figure 4:**
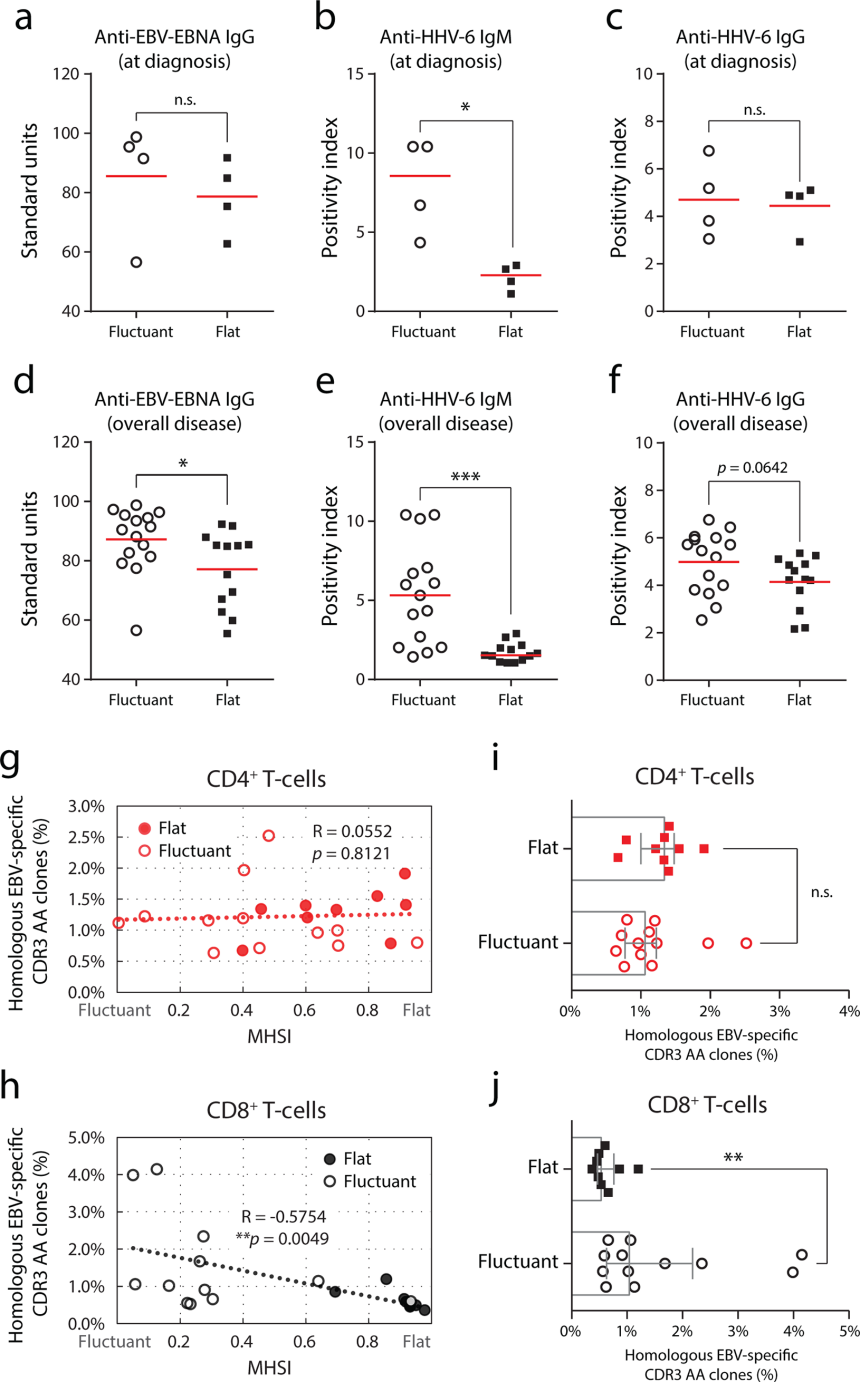
Abundance of the homologous EBV-specific CDR3 AA clonotypes reflects repertoire fluctuation. **(a, d)** Standard units ofIgG class antibodies against the EBV–EBNA epitope in serum. **(b, e)** Positivity index of IgM class anti-HHV-6 antibodies in serum. **(c, f)** Positivity index of IgG class anti-HHV-6 antibodies in serum. **(g, h)** Correlation between repertoire fluctuation and the average abundance of homologous EBV-specific CDR3 AA clonotypes for each sampling interval. Pearson correlation was calculated. **(i, j)** Abundance of homologous EBV-specific CDR3 AA clonotypes for “flat/fluctuant” patients. Bars show the medians with interquartile ranges. Statistics based on Mann-Whitney *U*-test.

The serological immune response to herpes-viruses thought to be reactivated in most Asian DRESS patients coinciding the persistence of the syndrome^10^. As expected, assessment of anti-viral antibodies demonstrated a substantial elevation of IgM anti-HHV-6 antibodies in “fluctuant” patients, compared to the “flat” patients, at the time of diagnosis (Figure 4B). Furthermore, we found that the IgG antibodies to EBV–EBNA and HHV-6, and IgM antibodies to HHV-6, from the samples taken over the disease were also elevated in the “fluctuant” patients (Figure 4D-F). These data strongly implicated higher elicited responses to a virus-challenge for “fluctuant” patients than that for “flat” patients.

Since the anti-herpes virus T-cell responses are primarily responsible for the pathogenesis of DRESS and guide the categorization of severe/mild patients^5^, the sequences of CDR3 AA clonotypes in our datasets were aligned with the EBV-specific TCR CDR3 sequences available in public resource. The average percentage of these homologous clones from 2 adjacent sampling was calculated as the anti-EBV cellular response in this phase. The proportion of CD4^+^ homologous clones was almost independent of the reformation of repertoires (Figure 4G), indicating that the CD4^+^ helper response may not the driving force in repertoire reformation. In contrast, we found that these homologous clones in the CD8^+^ repertoire positively correlated with repertoire fluctuation (Figure 4H), providing strong evidence that expansion of EBV-specific CD8^+^ T-cells may contribute to the marked repertoire fluctuation during DRESS progression. When comparing the proportion of homologous clones in the “flat” and “fluctuant” groups, an increased percent only in the CD8^+^ repertoire was identified for “fluctuant” patients (Figure 4I, J). Taken together, this substantial difference reinforced our observation that persistent arising of EBV-specific CD8^+^ clones may be a driving force in repertoire fluctuation.

### Spontaneous onset of autoimmune disease in DRESS-cured “fluctuant” patients

An interesting observation is the spontaneous onset of autoimmune disease in DRESS-cured “fluctuant” patients. A 19-year-old woman in the “fluctuant” group was re-admitted to our hospital at 2 months after the primary onset of DRESS, due to the occurrence of alopecia and leukoplakia. The patient had no history of any autoimmune disease. Physical and laboratory examinations supported the diagnosis of immune reconstitution inflammatory syndrome (IRIS). Thyroid function appeared to be compromised (free triiodothyronine: 2.06 pmol/L [normal: 2.8–7.1 pmol/L], free thyroxine: 7.06 pmol/L [normal: 12–22 pmol/L]), and increased titer of auto-antibody to thyroid peroxidase (TPO). Citing the anxiety of unwanted complications, this patient declined further sequencing to be performed. Therefore, whether the repertoire fluctuation could serve as an indicator for suffering autoimmune disease warrants further follow-up investigations.

## Discussion

Our repertoire characterization illustrated the details of dynamic repertoire reformation and found that, during the course of DRESS progression, the fluctuation in the CD8^+^ repertoire was positively correlated with the severity of clinical symptoms and with the magnitude of the anti-EBV immune response. To the best of our knowledge, this is the first identification of a biomarker for dynamic monitoring of the immune repertoire that could independently reflect the clinical severity of patients with DRESS. The dynamic fluctuation of the circulating CD8^+^ T-cell repertoire may contribute to the established knowledge on antiviral immunity-mediated DRESS pathogenicity. Therefore, monitoring of the CD8^+^ T-cell repertoire may provide clinical benefits in categorization of high-risk patients and guidance for personalized care. Furthermore, enhancement of the stability of dominant CD8^+^ T-cell clones in peripheral repertoire, and, suppression of the anti-herpes virus immune response may be a novel strategy for improving the efficacy of immunotherapy in DRESS.

The proportion and cytotoxic activity of reactive CD8^+^ T-cells is associated with the severity of clinical features *in vivo*. Patients with severe cutaneous or visceral lesions exhibit greater accumulation of CD8^+^ T-lymphocytes that secrete TNF-α, IFN-λ, and interleukin-2 than did patients with milder symptoms^5^. In agreement with our initial analysis, we also identified a higher proportion of CD8^+^ homologous EBV-specific CDR3 AA clones in patients with severe symptoms, suggesting that our strategy provided consistent results and was feasible. Interestingly, according to our longitudinally sampled repertoire, fluctuations in the CD8^+^ repertoire were associated with the severity of clinical features. Since the repertoire fluctuation mainly mirrored the egression and regression of dominant clones, these observations strongly implied that the reformation of the CD8^+^ repertoire, i.e., the continued production of newly proliferated CD8^+^ T-cell clones may also responsible for the maintenance of DRESS-related injury.

Studies in autoimmune disease continues to support the hypothesis that the initial tissue damage during a primary response can lead to the acquisition of novel auto-antigens, priming of quiescent self-reactive lymphocytes, and eventual secondary tissue damage. This scenario, referred to as “epitope spreading”, is thought to provide neo-autoreactive T-cells and maintain the inflammatory milieu^11^. Lehmann et al^12^ demonstrated that, after immunization with an immunodominant MBP 1–11 epitope, the restricted repertoire broadens to include the cryptic epitopes p35–47, p81–100, and p121–140 over time. The acquisition of newly autoreactive T-cells invariantly correlates with disease progression/relapse, and pre-tolerization to spreading epitope leads to improved clinical scores and milder demyelination^13^. Moreover, serial changes in newly autoreactive CD4^+^ T-cells have also been identified during the progression of human multiple sclerosis in an HLA-DR-restricted manner, indicating that the inherent instability of self-reactive immunity may contribute to the persistent damage observed in these patients^14^.

Our data led us to speculate that, in addition to the cross-reactivity of the antiviral response, the severity for DRESS patients could be alternatively attributed to the continuous spreading of self-reactive dominant CD8^+^ T-cell clones. First, the pathogenic spreading from virus- to self-epitopes has been verified in several studies^15^, and, the immunosuppression^16^ and frequent reactivation of herpes virus^10^ in the early stages of the disease can provide favorable conditions that trigger this process. Second, the decreased number of Tregs is accompanied by exacerbated symptoms and malfunctioning of multiple organs^17^, suggesting that a poor-restricted immune balance could facilitate visceral lesions. Third, highly expanded, self-reactive, dominant clones may experience functional tolerance^18^ and apoptotic deletion^19^ in the peripheral niche, leaving freshly primed, quiescent auto-reactive T-cells to fully differentiate and sustain disease^20^. Fourth, Cha et al. demonstrated that, for cancer patients, favorable prognoses are associated with the maintenance of pre-primed CD8^+^dominant clones, equivalent to the maintenance of anti-tumor TCR-specificity^21^. Accordingly, we infer that the gain/loss of pre-primed dominant clones is equivalent to the gain/loss of dominant TCR specificities, and, the spreading of dominant clones would lead to the spreading of dominant TCR-specificities. Finally, commencement of corticosteroid^10^, IVIG^22^, and/or other immunosuppressant^23^ will generally cause alleviation of clinical symptoms and abnormalities in laboratory tests over the course of disease.

Therefore, in combination with clinical evidence supporting the sequential reactivation of various human herpesviruses^24^,^25^, we propose a model in which persistent antiviral cross-reactivity triggers and maintains the release/presentation of self-immunogens. As a result, *de novo* primed quiescent self-reactive CD8^+^ T-cells give rise to dominant pathogenic clones, which in turn reinforce secondary tissue injury and, the sequential release of novel self-immunogens in a positive-feedback mechanism. Hence, the fluctuation of the CD8^+^ T-cell repertoire, determined by the instability of dominant clones, represents the occurrence of repertoire spreading in sustaining tissue injury that resulted from the initial insult in patients with DRESS. Although the detailed mechanism remains unclear, the clinical relevance of the fluctuant CD8^+^ T-cell repertoires supports the notion that herpesvirus-mediated spreading reactivation is an alternate mechanism responsible for the pathogenicity of DRESS.

## Methods

### Patients and diagnostic criteria

The study was approved by the Ethics Committee of the Southwest Hospital of Third Military Medicine University (identifier: KY201230) according to the ethical guideline of Helsinki Declaration. All patients gave written informed consent prior this trial. Patients were diagnosed with DRESS according to the criteria proposed by Bocquet et al^26^, then, were administered an equivalent therapy and received supportive care according to standard management^27^. Karnofsky performance scores (KPS)^28^ were determined to evaluate patients’ general well-being and quality of life at diagnosis. The RegiSCAR scoring system, which primarily serves as diagnostic criteria^29^, was used to assess general performance, skin lesions, visceral involvement, and laboratory examinations for patients. 10 mL of peripheral blood were collected from 8 patients at an interval of about 10-days from the time of clinical diagnosis (Supplementary Figure S1). The CD4^+^/CD8^+^ subsets were then isolated and sequenced. Depending on the duration of the disease, samples were obtained at 3 (4/8 patients), 4 (3/8 patients), or 5 (1/8 patients) time points from each individual. We also collected 10 mL of peripheral blood from 28 healthy donors who had a history of culprit exposure, but did not experience hypersensitivity. Summary of the patients’ clinical and biological characteristics is given in Supplementary Table S1. Human leukocyte antigen (HLA) typing are provided in Supplementary Tables S2. No associations were observed between HLA typing, offending drugs, age, or gender and clinical severity of DRESS.

### Isolation of peripheral blood mononuclear cells (PBMCs) and cell sorting

To separate the CD4^+^/CD8^+^ T-cell fractions from patients’ sample, PBMCs were isolated by density-gradient centrifugation (Percoll, GE Healthcare, Little Chalfont, UK) according to standard protocols. CD3^+^CD4^+^ and CD3^+^CD8^+^ cells were sorted using a FACSAria Cell Sorter (BD Biosciences, San Jose, CA, USA). Sorted cells were lysed in TRIzol (Invitrogen, Carlsbad, CA, USA). At the time of diagnosis, an additional 10 mL of fresh peripheral blood was used to isolate mononuclear cells, which were immediately lysed in TRIzol. PBMC from healthy donors were isolated and lysed in TRIzol directly.

### ELISA assays

Quantitative measurement of HHV-6-specific IgM and IgG antibodies (VIDIA, Jesenice, Czech Republic) and EBV–EBNA-specific IgG antibodies (Abcam, Cambridge, UK) in the sera of patients with DRESS were determined according to the manufacturer’s instructions. Standard units of anti-EBV–EBNA antibodies were as follows: >11, positive; 9–11, grey zone; <9, negative. The positivity index of anti-HHV-6 IgM antibodies was as follows: >3.00, (+++) positive; 2.01–3.00, (++) positive; 1.11–2.00, (+) positive; 0.90–1.10, (−/+); 0.90, negative. The positivity index of anti-HHV-6 IgG antibodies was as follows: >4.00, (+++) positive; 2.01–4.00, (++) positive; 1.11–2.00, (+) positive; 0.90–1.10, (−/+); <0.90, negative.

### Library construction and sequencing

Sequencing of the *TCRB* CDR3-regions was performed on Ion Torrent PGM platform (Life Technologies, Carlsbad, CA, USA). Briefly, total RNA was extracted and reverse transcribed into cDNA with a universal primer (RT primer: 5′-ATCTCTGCTTCTGATGGCTCA-3′). Multiplex PCR was employed to amplify the rearranged *TCRB* CDR3-region. A set of forward primers, each specific to one or a set of functional TCR V-beta segments, and universal reverse primers specific to the constant region of *TCRB,* were used to generate amplicons that covered the entire CDR3 region. Purified products were sequenced on a PGM platform. The FASTQ files obtained by sequencing were imported to the MATLAB 2014a (MathWork, Natick, MA, USA) for sequence alignment. The *TCRB* CDR3 region was determined according to the International ImMunoGeneTics (IMGT) collaboration^30^. Resulting sequencing data were further analyzed with MATLAB 2014a using manual scripts (Summary of sequencing in Supplementary Table S3).

### Statistical analysis

To quantify the similarity between TCR repertoires, we adopted the Morisita–Horn similarity index (MHSI), based on the percentage and homogeneity of shared clones within two repertoires^31^. A value from zero (no overlap for two repertoires) to one (identical overlap for two repertoire) was calculated for each pair of repertoires. All data were analyzed using two-tailed tests, and differences with *P*-values of less than 0.05 were considered statistically significance, unless otherwise specified.

## Supplementary Material

Supplementary Information Supplementary Information
