# Supplementary material for: Association of CD8+ T lymphocyte repertoire spreading with the severity of DRESS syndrome
Source: Sci Rep. 2015 Apr 23;5:9913. doi: 10.1038/srep09913 (PMC4649994; doi:10.1038/srep09913)
Supplement: Supplementary Information [file srep09913-s1.pdf]

Supplementary Materials

Association of CD8$^+$ T lymphocyte repertoire spreading with the severity of DRESS syndrome

Jun Niu[1#], Qingzhu Jia[2,3#], Qingshan Ni[2,3], Yi Yang[2,3], Gang Chen[2,3], Xichuan Yang[1], Zhifang Zhai[1], Haili Yu[2,3], Peng Guan[2,3], Regina Lin[4], Zhiqiang Song[1], Qi-Jing Li[3,4], Fei Hao [1]*, Hua Zhong[1]*, Ying Wan[2,3]*

[1]Department of Dermatology, Southwest Hospital, Third Military Medical University, Chongqing, China

[2]Biomedical Analysis Center, Third Military Medical University, Chongqing, China

[3]Chongqing Key Laboratory of Cytomics, Chongqing, China

[4] Department of Immunology, Duke University Medical Center, Durham, North Carolina, USA

[#]Jun Niu and Qingzhu Jia contributed equally as co-first authors.

*Fei Hao, Hua Zhong, and Ying Wan are co-corresponding authors.

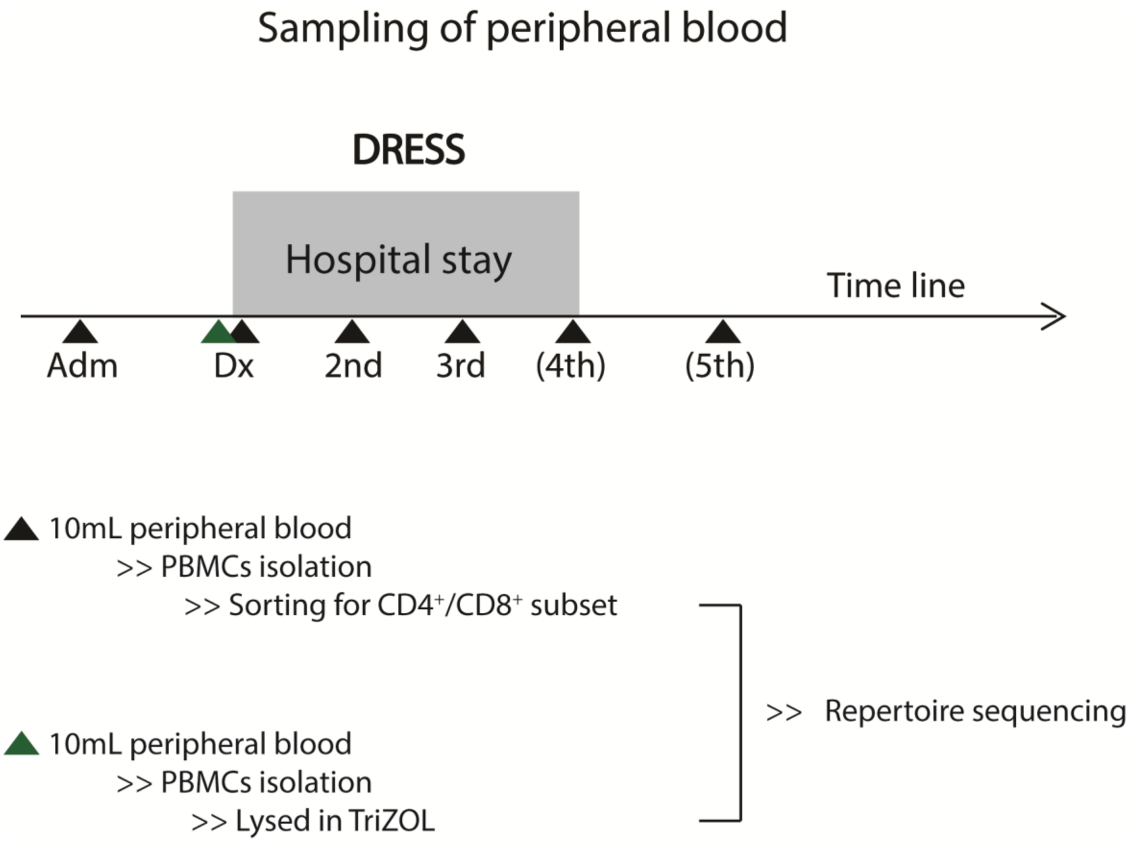

Figure-S1. Patients and sample procurement

Schematic view of collection of blood samples from DRESS patients. Grey areas show the duration of hospital stay. Adm, administration of the culprit drug; Dx, diagnosis; brackets, sampling from only certain patients. Black triangle, sampling for sorting of $CD4^{+}/CD8^{+}$ T-cell subset. Green triangle, sampling for PBMC sequencing.

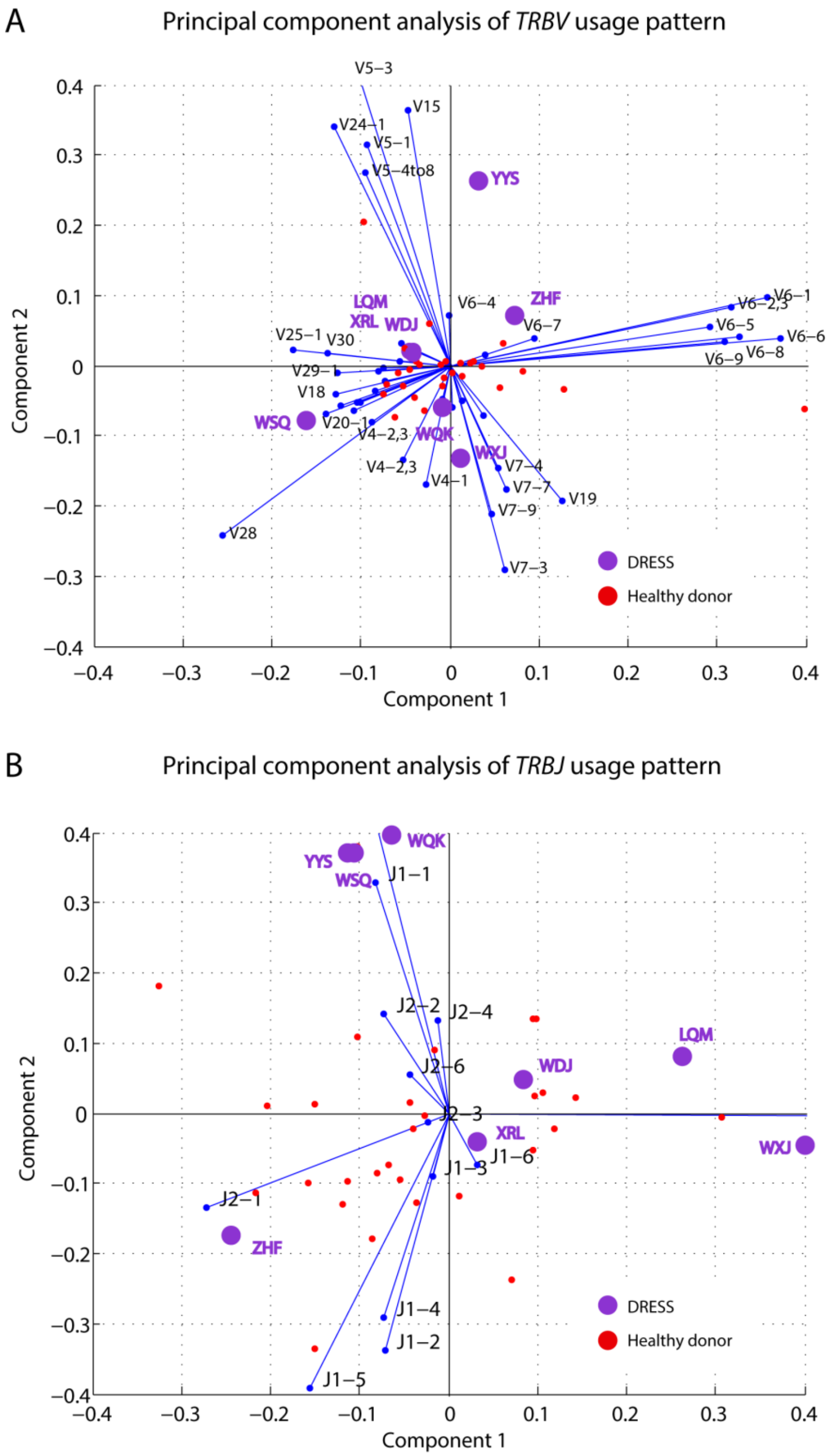

Figure-S2. *TRBV* and *TRBJ* segment usage patterns

Principal component analysis showed the overall usage patterns of **(A)***TRBV* and **(B)***TRBJ* segments for patients with DRESS and healthy donors. The scatterplot helps to visualize both the principal component coefficients for each gene segment and the principal component scores for each patient. The purple and red dots represent the PBMC repertoires from patients with DRESS and from healthy donors, respectively. Parts of the label for the V-beta segment were deleted to improve visual clarity.

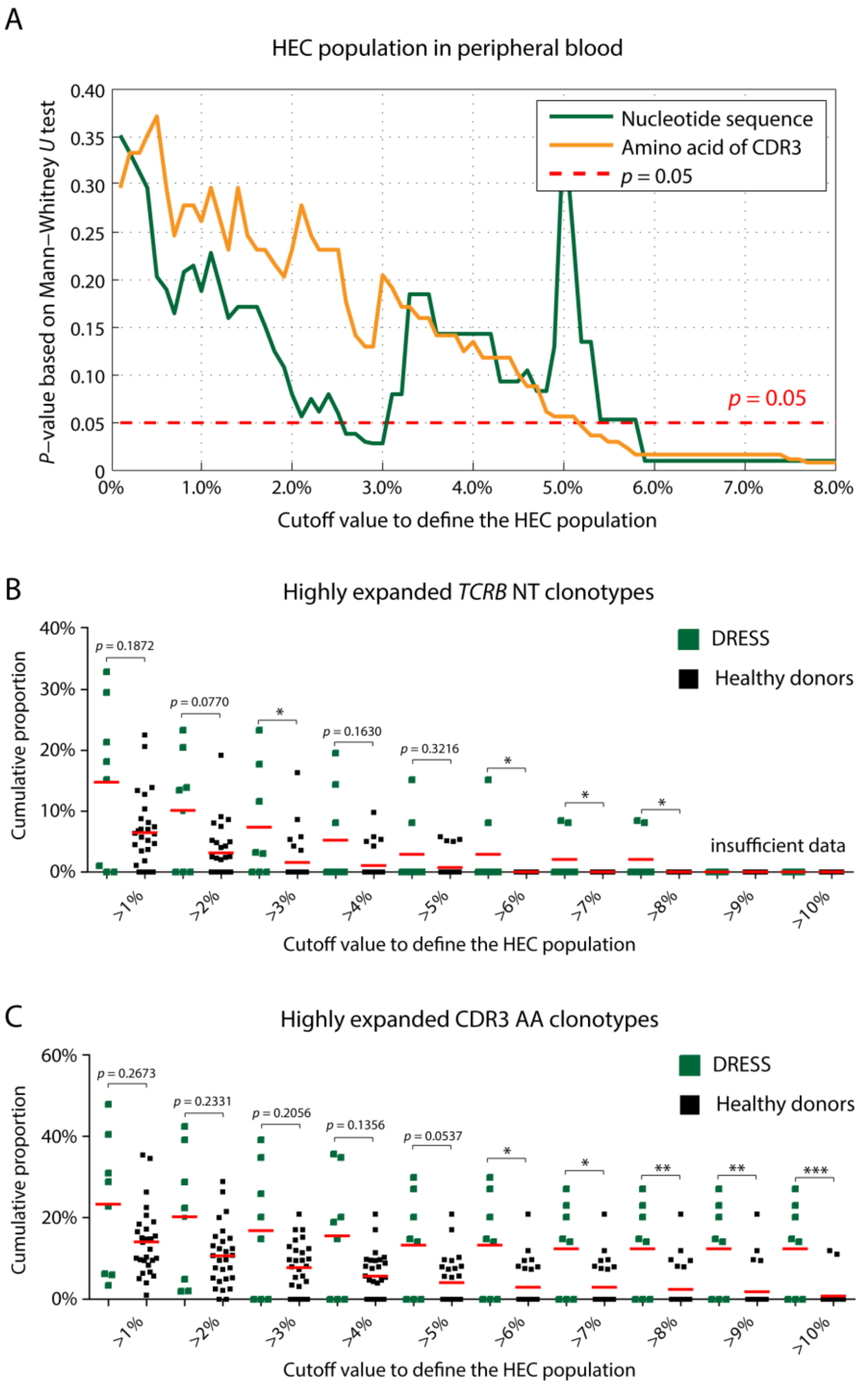

Figure-S3. Accumulation of highly expanded clonotypes in DRESS patients

(A) Mann–Whitney *U*-tests were used to compare the proportions of highly expanded clones (HECs) in patients and healthy donors. Significance of increased HECs in DRESS patients is represented as the *p*-value of the hypothesis test against the cutoff percentage that defined this HEC population. Calculation was performed for both the *TCRB* NT clones (defined as the T-cell population in which the nucleotide sequences in the entire *TCRB* transcript were identical) and for the CDR3 AA clones (defined as the T-cell population in which the amino acid sequence in the CDR3 region were identical). **(B, C)** Comparison of the proportion of expanded clones for both *TCRB* NT and CDR3 AA clones. Cutoff values for defining the HEC population are indicated on the horizontal axis (at intervals at 1%). Red bars, median values. Statistics based on Mann–Whitney *U*-tests.

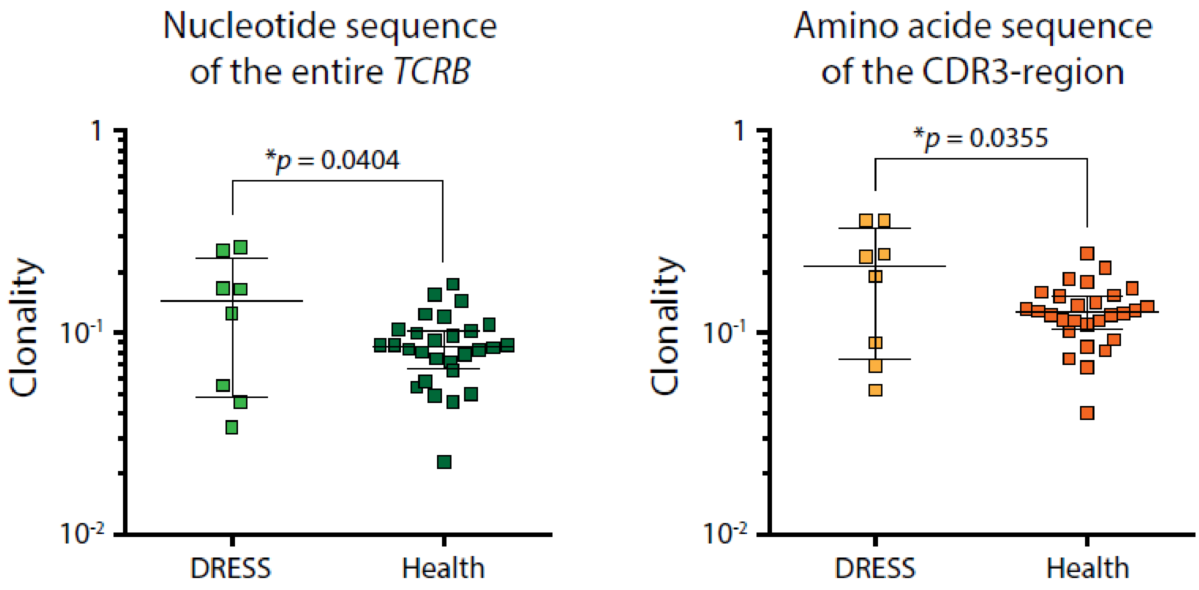

Figure-S4. Oligo-clonality of repertoire from peripheral blood

Dot plots showed the clonality index for DRESS cohort and condition-matched healthy donors. Higher index value, significant oligo-clonality; lower index value, prefer to poly-clonality. Left panel, for nucleotide sequence in the entire TCRB locus; right panel, for the amino acid sequence in CDR3 region.

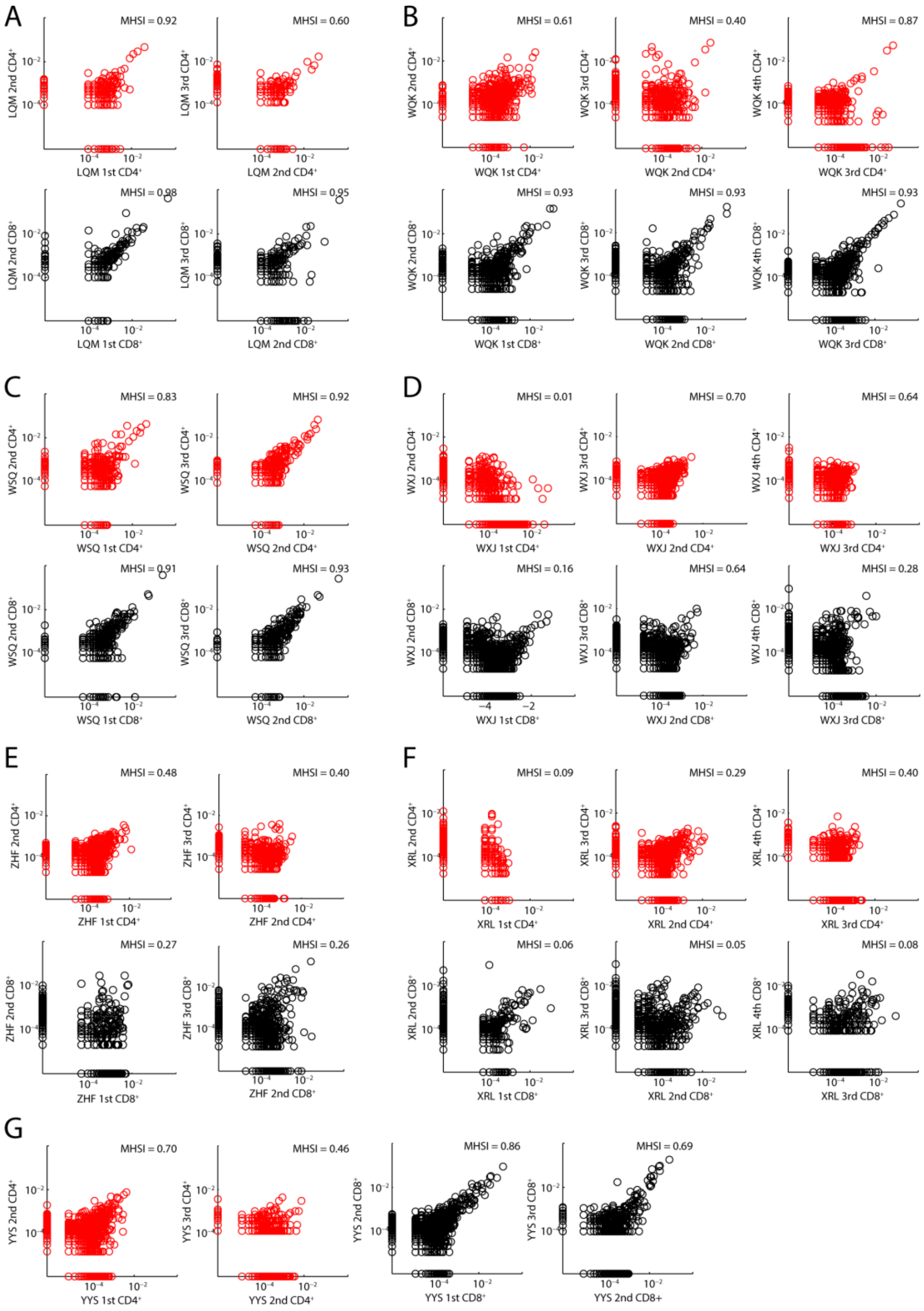

Figure-S5. Repertoire reformation during course of DRESS

Scatterplot showing the dynamic repertoire change for $CD4^+$ (red dots) and $CD8^+$ (black dots) T-cell clones. **(A-G)** Each dot represents a unique CDR3 AA clone and the corresponding abundance in 2 blood samples from adjacent time points. The dots on the axis indicate the clonotypes detected within a single repertoire. The MHSI is shown in the upper right corner for each panel.

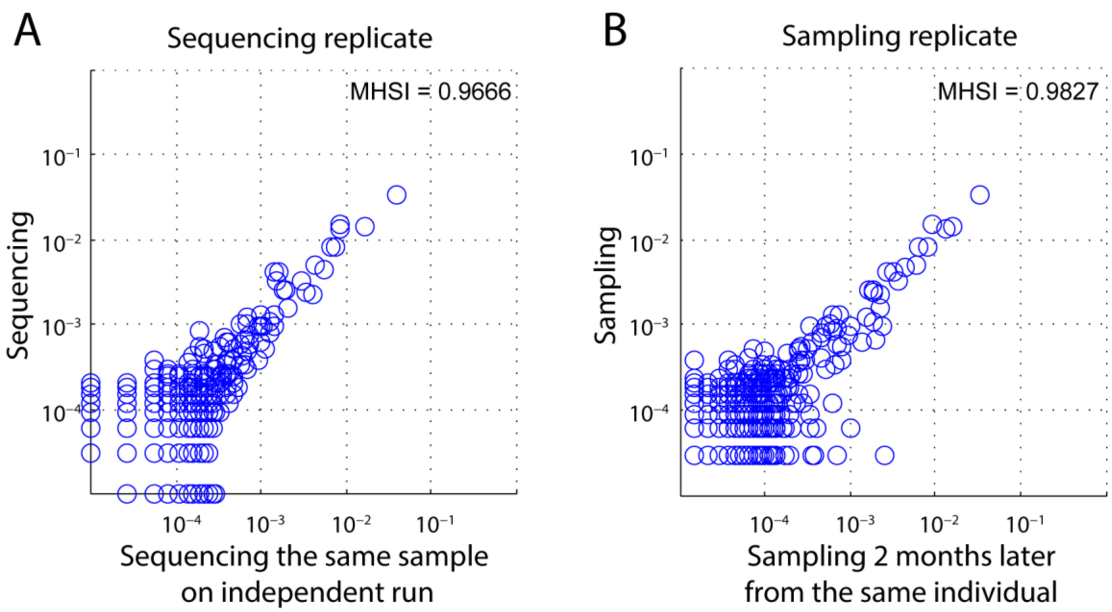

Figure-S6. Feasibility of sequencing strategy

Quality-control experiment to validate the reproducibility of our sequencing strategy. **(A)** Reproducibility of sequencing replicate. Peripheral blood mononuclear cells (PBMCs) were isolated from peripheral blood (10mL) obtained from a healthy donor for library construction. This library was sequenced twice independently to show the reproducibility of CDR3 AA clones. **(B)** Reproducibility of sampling replicates. PBMCs from an additional 10mL of peripheral blood were obtained from the same healthy donor at 57 days after the first sampling. The library was constructed and sequenced using the same protocol. The donor had no evidence of any immune-challenge, such as infections or medication, during this sampling interval. MHSI was calculated and shown in the upper right corner for each panel.

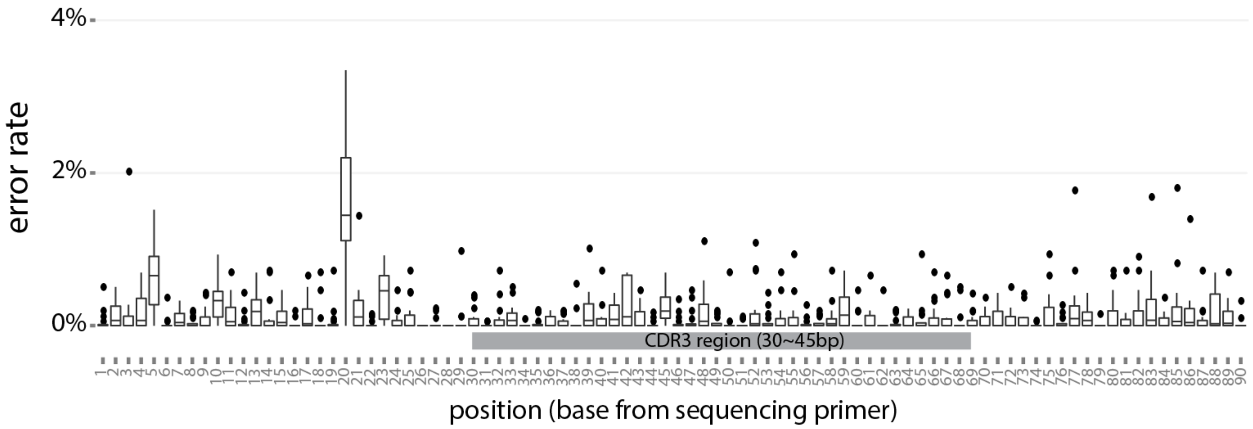

Figure-S7. Estimation of sequencing error rate

Error rate was estimated for sequencing reads covering the entire CDR3 region. 56 distinct PCR products were cloned into T-vector as standard template, and, sequenced independently according to identical protocol in this project. Box plot illustrated the distribution of error rate for each standard template in certain position. The overall error rate was 0.16% in the sequencing reads and 0.14% in CDR3 region.

Table-S1. Clinical and biological features of eight DRESS patients (at the time point of diagnosis).

	LQM	WDJ	WQK	WSQ	WXJ	XRL	YYS	ZHF
**Age/gender**	40/F	47/M	50/M	71/M	51/F	19/F	73/M	63/F
**Drugs**	1	2	1	1	3	4	5	6
**Fever ≥ 38.5°C**	Yes	Yes	Yes	Yes	Yes	Yes	Yes	Yes
**Enlarged lymph nodes**	Yes	Yes	Yes	No/U	Yes	Yes	No/U	Yes
**Eosinophilia**	Yes	Yes	No/U	Yes	Yes	Yes	Yes	No/U
**Atypical lymphocytes**	Yes	No/U	No/U	No/U	No/U	No/U	No/U	No/U
**Skin involvement**								
**Skin rash extent (% of skin area)**	>50%	>50%	<50%	>50%	>50%	>50%	>50%	>50%
**Skin rash suggesting DRESS**	Yes	Yes	Yes	Yes	Yes	Yes	Yes	Yes
**Biopsy suggesting DRESS**	Yes	No/U	No/U	No/U	No/U	No/U	No/U	No/U
**Organ involvement**								
**Liver**	Yes	Yes	Yes	Yes	Yes	Yes	Yes	Yes
**Kidney**	No/U	Yes	Yes	No/U	No/U	No/U	No/U	No/U
**Muscle/heart**	No/U	No/U	Yes	No/U	Yes	No/U	Yes	No/U
**Pancreas**	No/U	No/U	No/U	No/U	No/U	No/U	No/U	No/U
**Other organ**	Yes	Yes	No/U	No/U	Yes	Yes	No/U	No/U
**Resolution ≥ 15 days**	Yes	Yes	Yes	Yes	Yes	Yes	Yes	Yes
**Evaluation of other potential causes**								
**Antinuclear antibody**	Yes	No/U	No/U	No/U	No/U	No/U	No/U	No/U
**Blood culture**	No/U	No/U	No/U	No/U	No/U	No/U	No/U	No/U
**Serology for HAV/HBV/HCV**	No/U	No/U	No/U	No/U	No/U	No/U	No/U	No/U
**Chlamydia/mycoplasma**	No/U	No/U	No/U	No/U	No/U	No/U	No/U	No/U
**RegiSCAR score**	7	7	5	5	6	7	5	7
**Karnofsky score**	70%	40%	70%	70%	50%	40%	60%	40%

Drugs:
1. Antibiotics
2. Allopurinol
3. Traditional Chinese Medicine
4. Lamotrigine
5. Carbamazepine
6. Sulfasalazine

Table-S2. Human leukocyte antigen (HLA) typing of patients with DRESS

Patient	HLA-A*	HLA-B*	HLA-C*	HLA-DRB1*	HLA-DQB1*	HLA-DPB1*
**LQM**	A*02:03:01<br>A*11:01:01	B*13:01:01<br>B*15:02:01	C*03:04:01<br>C*08:01:01G	DRB1*16:02:01<br>DRB1*16:02:01	DQB1*05:02:01<br>DQB1*05:02:01	DPB1*05:01:01G<br>DPB1*05:01:01G
**WDJ**	A*24:02<br>A*30:01:01	B*13:02:01<br>B*58:01:01	C*03:02<br>C*06:02:01G	DRB1*07:01:01<br>DRB1*13:02:01	DQB1*02:01:01<br>DQB1*06:09:01	DPB1*02:01:02G<br>DPB1*02:01:02G
**WQK**	A*24:02<br>A*24:02	B*15:27:01<br>B*40:01	C*03:04:01<br>C*04:01:01G	DRB1*04:05:01<br>DRB1*11:01:01	DQB1*03:01:01<br>DQB1*04:01:01	DPB1*05:01:01G<br>DPB1*13:01:01G
**WSQ**	A*02:07:01<br>A*03:01:01	B*46:01:01<br>B*51:01:01	C*01:02:01G<br>C*14:02:01	DRB1*14:04<br>DRB1*16:02:01	DQB1*05:02:01<br>DQB1*05:03:01	DPB1*02:01:02G<br>DPB1*05:01:01G
**WXJ**	A*02:07:01<br>A*24:02	B*15:02:01<br>B*46:01:01	C*01:02:01G<br>C*08:01:01G	DRB1*09:01:02<br>DRB1*12:02:01	DQB1*03:01:01<br>DQB1*03:03:02	DPB1*02:02<br>DPB1*02:02
**XRL**	A*11:01:01<br>A*11:01:01	B*13:01:01<br>B*13:01:01	C*03:04:01<br>C*03:04:01	DRB1*15:01<br>DRB1*15:01	DQB1*06:01:01<br>DQB1*06:01:01	DPB1*05:01:01G<br>DPB1*14:01
**YYS**	A*11:01:01<br>A*24:02	B*08:01<br>B*13:01:01	C*03:04:01<br>C*07:02:01G	DRB1*09:01:02<br>DRB1*13:02:01	DQB1*03:03:02<br>DQB1*06:04:01	DPB1*02:01:02G<br>DPB1*05:01:01G
**ZHF**	A*11:01:01<br>A*11:01:01	B*13:01:01<br>B*15:02:01	C*07:02:01G<br>C*08:01:01G	DRB1*12:02:01<br>DRB1*13:12:01	DQB1*03:01:01<br>DQB1*03:01:01	DPB1*05:01:01G<br>DPB1*05:01:01G

Table-S3. Summary of sequencing

Patients	Sampling	Compartment	Reads	Unique TCRB NT clones	Unique CDR3 AA clones
**LQM**	**First**	CD4+	10205	7803	5796
		CD8+	10449	3218	2292
		PBMC	21063	17272	15538
	**Second**	CD4+	9513	7291	5585
		CD8+	10274	2835	1917
	**Third**	CD4+	7464	6096	4866
		CD8+	16012	7528	5244
**WDJ**	**First**	CD4+	49196	28685	16601
		CD8+	63599	20810	9435
		PBMC	32132	24513	21314
	**Second**	CD4+	64836	44988	32562
		CD8+	95138	32449	17988
	**Third**	CD4+	42660	32836	25229
		CD8+	41827	14816	9761
	**Forth**	CD4+	58632	40017	29664
		CD8+	42551	9884	3866
	**Fifth**	CD4+	126374	81816	63147
		CD8+	36184	12384	8723
**WQK**	**First**	CD4+	42341	25907	20091
		CD8+	47034	18895	14060
		PBMC	30088	17351	16063
	**Second**	CD4+	40572	29517	22221
		CD8+	36808	11980	6945
	**Third**	CD4+	39755	19505	12508
		CD8+	48155	13688	7086
	**Forth**	CD4+	60794	38604	23073
		CD8+	53545	12646	6481
**WSQ**	**First**	CD4+	15277	10725	7941
		CD8+	20236	10911	8850
		PBMC	17558	6916	4679
	**Second**	CD4+	17264	11294	8635
		CD8+	17776	5496	3756
	**Third**	CD4+	11596	7406	5543
		CD8+	15560	5576	3790
**WXJ**	**First**	CD4+	67134	20205	9023
		CD8+	85472	27975	14450
		PBMC	54080	20716	16394
	**Second**	CD4+	68129	46737	29525
		CD8+	55181	38607	25906
	**Third**	CD4+	48242	37826	26539
		CD8+	54170	27183	12719
	**Forth**	CD4+	62289	53138	43651
		CD8+	71965	36201	26143
**XRL**	**First**	CD4+	11621	11251	10420
		CD8+	14824	11071	9549
		PBMC	42873	37427	34533
	**Second**	CD4+	61152	44894	35950

		CD8+	94622	56998	44575
	**Third**	CD4+	63636	51971	42574
		CD8+	65379	22589	12545
	**Forth**	CD4+	11221	9853	7993
		CD8+	12760	9454	6663
**YYS**	**First**	CD4+	106119	57741	26726
		CD8+	81671	23756	12385
		PBMC	56797	21731	18792
	**Second**	CD4+	73220	34889	14722
		CD8+	106823	35950	15790
	**Third**	CD4+	8290	7768	6821
		CD8+	11642	3070	2164
**ZHF**	**First**	CD4+	40048	29016	21934
		CD8+	18893	5431	2339
		PBMC	26122	15777	13086
	**Second**	CD4+	52990	36918	23675
		CD8+	47756	23512	12734
	**Third**	CD4+	38631	32496	26602
		CD8+	68936	17108	11061
**Healthy Donor 01**		PBMC	83356	56904	44612
**Healthy Donor 02**		PBMC	89660	62263	49785
**Healthy Donor 03**		PBMC	72983	61195	51862
**Healthy Donor 04**		PBMC	95828	61886	46700
**Healthy Donor 05**		PBMC	88744	55842	38909
**Healthy Donor 06**		PBMC	97239	73139	60170
**Healthy Donor 07**		PBMC	50024	36334	30728
**Healthy Donor 08**		PBMC	69501	47925	36702
**Healthy Donor 09**		PBMC	64891	46845	36910
**Healthy Donor 10**		PBMC	69883	48493	40772
**Healthy Donor 11**		PBMC	73451	50653	40555
**Healthy Donor 12**		PBMC	32074	24937	21588
**Healthy Donor 13**		PBMC	60295	32517	26047
**Healthy Donor 14**		PBMC	61029	43031	35595
**Healthy Donor 15**		PBMC	59184	30824	26689
**Healthy Donor 16**		PBMC	67278	44504	36794
**Healthy Donor 17**		PBMC	60091	41444	32231
**Healthy Donor 18**		PBMC	65424	45855	35876
**Healthy Donor 19**		PBMC	51556	41619	35818
**Healthy Donor 20**		PBMC	80494	48362	39947
**Healthy Donor 21**		PBMC	59422	39608	31930
**Healthy Donor 22**		PBMC	61518	36000	27713
**Healthy Donor 23**		PBMC	46552	30298	25267
**Healthy Donor 24**		PBMC	35508	22306	17584
**Healthy Donor 25**		PBMC	55941	26955	22165
**Healthy Donor 26**		PBMC	15743	10578	9321
**Healthy Donor 27**		PBMC	33172	23049	19368
**Healthy Donor 28**		PBMC	42544	28430	17399

Table-S4. Persistent and newly dominant clones (merged from TOP20 clone at each sampling).

Patient	Sampling					Persistent
**LQM**	**First**	**Second**	**Third**			**TCPI$_{20}$=20/32**
'CAAAWGPPSEQY'	0.325%	0.292%	0.156%			Yes
'CAISEGDSPYEQY'	0.124%	0.117%	0.256%			Yes
'CAISESTRNQPQH'	0.105%	0.146%	0.731%			Yes
'CASSAGELF'	1.034%	1.168%	0.768%			Yes
'CASSETSGSFGTGELF'	N.D.	N.D.	0.256%			
'CASSETTVHYEGAF'	1.771%	1.197%	1.980%			Yes
'CASSFASVGNEQF'	3.168%	2.073%	0.012%			Yes
'CASSFEAGTYGYT'	0.010%	1.392%	N.D.			
'CASSISENTEAF'	0.450%	0.399%	N.D.			
'CASSLAGSTDTQY'	2.909%	1.713%	0.006%			Yes
'CASSLEQTTRTDTQY'	0.488%	0.360%	0.181%			Yes
'CASSLETQY'	0.354%	0.467%	N.D.			
'CASSLGASYEQY'	44.789%	41.493%	33.163%			Yes
'CASSLGNEQF'	N.D.	N.D.	0.356%			
'CASSLGPPSEQY'	0.584%	0.623%	0.294%			Yes
'CASSLGQITDTQY'	0.412%	0.243%	N.D.			
'CASSLSGFGTDTQY'	0.517%	0.331%	N.D.			
'CASSLTFHQQETQY'	0.077%	0.068%	0.412%			Yes
'CASSNLGRPTYTPLSYEQY'	0.182%	0.944%	N.D.			
'CASSNPSFNQPQH'	0.364%	0.311%	0.250%			Yes
'CASSPGLPYEQY'	0.278%	0.195%	0.287%			Yes
'CASSQDEGLRHGYT'	N.D.	0.769%	0.337%			
'CASSQVASGSWGDTQY'	N.D.	0.370%	0.468%			
'CASSRGQRSTDTQY'	0.134%	0.234%	0.275%			Yes
'CASSRPSGTQY'	1.589%	1.879%	2.261%			Yes
'CASSSTDPGLPYNEQF'	0.268%	0.321%	0.250%			Yes
'CASSWGPPSEQY'	1.034%	0.526%	0.593%			Yes
'CASSYAYTDTQY'	0.488%	0.088%	N.D.			
'CASSYGQGNTEAF'	0.536%	8.585%	0.418%			Yes
'CASSYSPGDPLGAF'	0.900%	0.370%	N.D.			
'CASSYSRQGSHEQY'	0.679%	0.516%	0.269%			Yes
'CSNRTGTNSGNTIY'	0.651%	1.285%	0.281%			Yes
**WDJ**	**First**	**Second**	**Third**	**Fourth**	**Fifth**	**TCPI$_{20}$=27/68**
'CAGQEREAAGELF'	0.192%	0.092%	0.146%	N.D.	1.155%	
'CAISESAGETEQY'	0.000%	0.000%	0.000%	N.D.	0.627%	
'CAISESQTDTQY'	0.330%	0.179%	0.270%	0.099%	1.078%	Yes
'CASARDEQY'	0.024%	0.996%	1.657%	0.197%	1.354%	Yes
'CASEPRDRGWNEQF'	N.D.	N.D.	N.D.	0.282%	N.D.	
'CASGQGGDEQY'	0.035%	0.059%	0.100%	0.019%	1.727%	Yes
'CASGVTGGSTDTQY'	0.520%	0.075%	0.019%	N.D.	N.D.	

'CASREENTGELF'	2.115%	0.564%	0.416%	0.122%	0.006%	Yes
'CASRTGTSGSYEQY'	0.486%	0.060%	0.022%	N.D.	N.D.	
'CASSALGGILSYNEQF'	0.700%	0.077%	0.036%	0.012%	N.D.	
'CASSAWGNEQY'	0.006%	0.009%	0.002%	0.334%	0.503%	Yes
'CASSDINEQF'	0.014%	0.005%	N.D.	0.277%	N.D.	
'CASSEATSGSLGKETQY'	N.D.	0.562%	0.237%	N.D.	0.008%	
'CASSEEGGSGANVLT'	0.013%	0.057%	0.084%	N.D.	0.699%	
'CASSFEGPGELF'	N.D.	0.579%	0.739%	0.432%	0.229%	
'CASSFGTTNTGELF'	3.728%	1.639%	2.233%	0.219%	0.047%	Yes
'CASSFPSGSRDEQY'	N.D.	0.736%	2.149%	0.092%	0.033%	
'CASSFSSNQPQH'	0.582%	0.215%	0.182%	N.D.	0.403%	
'CASSHTGELF'	0.030%	0.117%	0.375%	0.026%	3.789%	Yes
'CASSIGAGSNQPQH'	N.D.	1.072%	1.592%	0.259%	0.055%	
'CASSLASETQY'	0.008%	2.487%	2.883%	0.879%	0.069%	Yes
'CASSLATAETQY'	0.420%	0.027%	0.029%	N.D.	N.D.	
'CASSLEQGTGELF'	0.489%	0.145%	0.201%	0.026%	0.003%	Yes
'CASSLGGQGYEQY'	N.D.	0.001%	N.D.	0.115%	3.073%	
'CASSLGLTGNEQF'	0.014%	0.269%	0.605%	0.047%	0.080%	Yes
'CASSLGMARSSYEQY'	N.D.	1.418%	1.078%	0.122%	0.265%	
'CASSLQQSQETQY'	0.681%	0.054%	0.031%	N.D.	N.D.	
'CASSLSGTNPWPYEQY'	0.011%	1.345%	2.785%	1.269%	0.354%	Yes
'CASSLTCQSSRRGYT'	0.560%	0.362%	0.244%	N.D.	N.D.	
'CASSLTINTGELF'	N.D.	0.022%	0.571%	0.115%	0.099%	
'CASSLVAGGGETQY'	N.D.	0.001%	N.D.	N.D.	0.879%	
'CASSLWGTSNIYGYT'	0.017%	0.748%	1.054%	0.188%	0.061%	Yes
'CASSPFYGGYGYEQY'	N.D.	0.502%	0.598%	0.026%	0.006%	
'CASSPGENEQF'	0.042%	0.013%	N.D.	0.505%	N.D.	
'CASSPGLAEYNEQF'	1.025%	0.293%	0.215%	0.230%	0.006%	Yes
'CASSPPGLVDNEQF'	0.190%	2.142%	3.287%	2.879%	1.362%	Yes
'CASSPRRYNEQF'	N.D.	0.628%	0.693%	N.D.	0.011%	
'CASSPWTTEAF'	0.050%	0.034%	0.084%	0.148%	2.777%	Yes
'CASSQDRATGELF'	0.019%	0.027%	0.091%	0.508%	0.279%	Yes
'CASSQDTGGNQPQH'	N.D.	1.948%	4.748%	0.338%	0.083%	
'CASSQESGNTEAF'	0.168%	0.171%	0.301%	1.873%	10.488%	Yes
'CASSQLQGSNQPQH'	N.D.	N.D.	N.D.	N.D.	0.553%	
'CASSSAGGPAETQY'	0.325%	0.471%	0.751%	0.101%	0.003%	Yes
'CASSSLIAGAGNEQF'	N.D.	0.048%	0.990%	0.367%	0.122%	
'CASSSSTEAF'	0.042%	0.017%	0.022%	0.226%	0.614%	Yes
'CASSTGPSFTGELF'	0.307%	0.057%	0.088%	0.160%	9.189%	Yes
'CASSTGTSGSEQY'	N.D.	N.D.	N.D.	0.296%	N.D.	
'CASSTRQGTYGYT'	5.035%	0.714%	0.404%	0.148%	0.146%	Yes
'CASSVGGLPGDEQF'	0.027%	0.011%	0.010%	N.D.	1.545%	
'CASSWKQSAGELF'	0.042%	0.063%	0.103%	0.402%	1.501%	Yes

'CASSWTSLPDEQY'	0.008%	N.D.	N.D.	0.282%	N.D.	
'CASSYFGQGSDTQY'	0.428%	0.054%	0.038%	0.169%	0.011%	Yes
'CASSYGGSTDTQY'	0.478%	0.225%	0.232%	N.D.	N.D.	
'CASSYLGQGSDIQY'	1.299%	0.107%	0.060%	0.016%	0.008%	Yes
'CASSYMAGQPQH'	0.992%	0.503%	0.292%	N.D.	0.025%	
'CASSYNPLVYEQY'	N.D.	0.520%	0.299%	N.D.	0.019%	
'CASSYRLGTGVSNQPQH'	N.D.	0.596%	0.098%	0.016%	0.006%	
'CASSYRTDPGELF'	0.362%	0.057%	0.012%	N.D.	N.D.	
'CASSYSLSFHNEQF'	N.D.	N.D.	N.D.	0.284%	N.D.	
'CASSYSRDNEQF'	N.D.	N.D.	N.D.	0.289%	N.D.	
'CASSYTPGESYEQY'	1.005%	0.279%	0.160%	N.D.	N.D.	
'CASSYVLNTEAF'	0.791%	0.148%	0.043%	N.D.	N.D.	
'CASTLDRERRSNQPQH'	0.002%	0.311%	1.050%	0.028%	1.998%	Yes
'CASTLGGNQPQH'	0.516%	0.164%	0.201%	0.054%	N.D.	
'CASTTSIYNEQF'	N.D.	0.003%	N.D.	0.303%	N.D.	
'CATKKGIEAF'	0.041%	7.662%	8.351%	0.369%	0.174%	Yes
'CATSRDTLAGAPQDTQY'	0.077%	0.037%	0.093%	0.414%	1.860%	Yes
'CSARPNEQY'	0.005%	0.015%	0.038%	N.D.	0.569%	
**WQK**	**First**	**Second**	**Third**	**Fourth**		**$TCPI_{20}$=25/31**
'CAIRAAGQGAYEQY'	0.848%	0.446%	0.723%	0.863%		Yes
'CASELVTGELF'	0.166%	0.312%	0.143%	0.106%		Yes
'CASGPPGFSGELF'	0.021%	0.342%	0.021%	N.D.		
'CASRGLAGTSDTQY'	0.753%	0.804%	0.239%	0.329%		Yes
'CASRLTGGSTEAF'	6.013%	2.434%	4.452%	4.988%		Yes
'CASRPPGSTYNEQF'	0.147%	0.206%	0.320%	0.317%		Yes
'CASRRRGAMETQY'	0.145%	0.149%	0.262%	0.472%		Yes
'CASSFPQVVGFPGYT'	N.D.	0.003%	0.370%	N.D.		
'CASSIGDLEQF'	0.168%	0.508%	0.075%	0.211%		Yes
'CASSLAYGQVGDGYT'	0.927%	0.937%	1.140%	1.156%		Yes
'CASSLLYFGRQNTGELF'	1.301%	1.869%	1.578%	1.686%		Yes
'CASSLRGYSNQPQH'	0.381%	0.196%	0.114%	N.D.		
'CASSPEGFETQY'	12.876%	13.516%	16.628%	24.914%		Yes
'CASSPGTVYGYT'	0.336%	0.269%	0.291%	0.190%		Yes
'CASSPPGGGPKNIQY'	1.059%	1.141%	1.038%	0.872%		Yes
'CASSPRAAGLTYEQY'	0.468%	0.144%	0.143%	0.080%		Yes
'CASSPTGATEAF'	2.407%	1.986%	2.878%	3.750%		Yes
'CASSQDRRDNQPQH'	8.498%	13.820%	7.978%	7.209%		Yes
'CASSQGGNLLEQF'	0.600%	1.739%	1.564%	1.860%		Yes
'CASSQGGNSYEQY'	0.742%	2.154%	2.091%	2.783%		Yes
'CASSRLYPGTDPDGYT'	0.070%	0.136%	0.071%	0.437%		Yes
'CASSYGNIQY'	0.755%	0.022%	N.D.	N.D.		
'CASSYGPGHGEQY'	0.436%	0.141%	0.328%	0.321%		Yes
'CASSYGTGRTGELF'	2.326%	2.059%	2.737%	2.344%		Yes

'CASSYSGYGYT'	0.032%	0.693%	0.033%	N.D.	
'CASSYYGGRADTQY'	0.389%	0.467%	1.094%	1.255%	Yes
'CATSDLVGDTPL'	N.D.	0.005%	1.560%	0.024%	
'CATSVSSGRHTGELF'	0.096%	0.120%	0.162%	0.433%	Yes
'CSARARRLAGGPDHAPDTQY'	0.525%	0.405%	0.451%	0.454%	Yes
'CSARDGIGRVGDQPQH'	0.427%	0.462%	0.469%	0.316%	Yes
'CSARVPRTGSTGNTIY'	0.512%	0.951%	0.557%	0.633%	Yes
**WSQ**	**First**	**Second**	**Third**		**$TCPI_{20}$=26/27**
'CASSAEGPGLDTDTQY'	0.080%	0.276%	0.604%		Yes
'CASSDGRRADEQF'	0.439%	0.619%	1.202%		Yes
'CASSFSAGTGDEQF'	0.581%	0.478%	0.964%		Yes
'CASSFSGSADTQY'	0.205%	0.782%	2.892%		Yes
'CASSGTGGAETQY'	0.547%	0.349%	0.231%		Yes
'CASSLEQGARTDTQY'	0.330%	0.456%	0.611%		Yes
'CASSLETRTGELF'	1.356%	N.D.	N.D.		
'CASSLGETQY'	0.450%	0.467%	0.572%		Yes
'CASSLGQGGAPQH'	0.626%	0.456%	0.379%		Yes
'CASSLQGYNEQF'	6.071%	4.135%	4.467%		Yes
'CASSLTLTGNTEAF'	25.379%	39.351%	26.877%		Yes
'CASSPWRDSGANVLT'	0.849%	0.675%	0.649%		Yes
'CASSQESAGSAYNEQF'	0.171%	0.467%	0.334%		Yes
'CASSQGSGQPQH'	0.182%	0.619%	0.649%		Yes
'CASSQKLTGELF'	0.456%	0.186%	0.418%		Yes
'CASSRGGWETQY'	0.626%	0.338%	1.015%		Yes
'CASSRLAGLADTQY'	1.190%	0.793%	1.459%		Yes
'CASSRQGAGPRETQY'	0.399%	0.596%	0.668%		Yes
'CASSRRSGSGNEQF'	1.008%	1.243%	1.324%		Yes
'CASSSLGTGGSRETQY'	0.604%	0.478%	0.656%		Yes
'CASSSWSSGTDTQY'	0.370%	0.208%	0.141%		Yes
'CASSVGTSGELF'	5.245%	4.962%	3.952%		Yes
'CASSVQGAGPRETQY'	1.213%	0.467%	0.790%		Yes
'CASSYPGQEA'	0.820%	0.664%	0.559%		Yes
'CASSYSLSVGEKLF'	0.228%	0.714%	0.810%		Yes
'CASWDHSYEQY'	0.273%	0.236%	0.810%		Yes
'CATDTSGGSSETQY'	0.427%	0.388%	0.276%		Yes
**WXJ**	**First**	**Second**	**Third**	**Fourth**	**$TCPI_{20}$=32/58**
'CAAETGGYEQY'	0.026%	0.005%	0.214%	N.D.	
'CASEPGLGYFAGELF'	0.371%	0.022%	0.035%	0.085%	Yes
'CASHGYSRADSGLF'	0.019%	0.312%	0.076%	N.D.	
'CASKKDLSTYEQY'	2.116%	0.544%	0.700%	0.449%	Yes
'CASKRGTGYTGELF'	0.098%	N.D.	0.006%	0.522%	
'CASLRGYREKLF'	N.D.	N.D.	N.D.	0.623%	
'CASNEDRRSSYNEQF'	0.468%	0.013%	N.D.	0.003%	

'CASNRGLSPYNEQF'	0.021%	N.D.	0.164%	N.D.	
'CASRFTSAGNEQF'	0.001%	0.045%	0.074%	0.809%	Yes
'CASRLGAAYGYT'	0.414%	0.043%	0.140%	0.427%	Yes
'CASRMTSGRGETQY'	0.002%	0.141%	0.024%	0.076%	Yes
'CASRPTDSHQPQH'	0.672%	0.105%	0.078%	0.361%	Yes
'CASRRLAGTYEQY'	0.621%	0.056%	0.159%	0.417%	Yes
'CASSALTGELF'	N.D.	N.D.	0.153%	N.D.	
'CASSDLSLAKNIQY'	N.D.	N.D.	N.D.	0.402%	
'CASSEDSMNTEAF'	0.051%	0.013%	0.151%	N.D.	
'CASSEGQGGETQY'	0.006%	0.076%	0.024%	0.755%	Yes
'CASSETSYNEQF'	0.008%	N.D.	0.162%	N.D.	
'CASSFLDREMETQY'	6.282%	0.533%	1.036%	0.447%	Yes
'CASSFNPGLAVYEQY'	0.502%	0.036%	0.109%	0.563%	Yes
'CASSFSDRTYSYEQY'	0.001%	0.152%	0.030%	0.018%	Yes
'CASSFTGGPVEQY'	0.006%	0.004%	0.166%	N.D.	
'CASSISGISYNEQF'	5.101%	0.265%	0.541%	0.705%	Yes
'CASSLASYEQY'	1.073%	0.134%	0.135%	0.710%	Yes
'CASSLDWGAGQPQH'	0.048%	0.007%	N.D.	0.552%	
'CASSLFTGGSGNTIY'	0.551%	0.027%	0.030%	0.093%	Yes
'CASSLGGGSSYEQY'	3.064%	0.199%	0.157%	0.357%	Yes
'CASSLGLPGNNEQF'	0.051%	0.002%	0.330%	N.D.	
'CASSLIQGGGNEQF'	0.029%	0.141%	0.026%	0.813%	Yes
'CASSLQGSNTEAF'	N.D.	N.D.	N.D.	8.663%	
'CASSLVAGPAYNEQF'	0.011%	0.167%	0.028%	0.040%	Yes
'CASSLVGGKSSYEQY'	0.448%	0.040%	0.042%	0.142%	Yes
'CASSPAGVGQPQH'	0.026%	0.004%	0.246%	N.D.	
'CASSPDQDYQPQH'	0.418%	0.042%	0.030%	0.003%	Yes
'CASSPGENEQF'	0.061%	N.D.	0.183%	0.001%	
'CASSPGGAPNEQF'	0.002%	0.013%	0.009%	0.364%	Yes
'CASSPGSGFAGELF'	0.004%	0.130%	0.009%	0.015%	Yes
'CASSPYSPGQGAHGQPQH'	N.D.	N.D.	N.D.	0.459%	
'CASSQAQGLGYT'	0.261%	0.060%	0.017%	0.721%	Yes
'CASSQEPGLAGVKDTQY'	0.834%	0.031%	0.009%	0.015%	Yes
'CASSQGLAGGNEQY'	0.020%	0.004%	0.153%	N.D.	
'CASSQGTGTATGELF'	0.026%	0.397%	0.022%	0.019%	Yes
'CASSQGTSGTTGELF'	0.006%	0.256%	0.044%	0.040%	Yes
'CASSRGDVQETQY'	0.001%	0.159%	0.028%	0.017%	Yes
'CASSRSAETQY'	N.D.	0.129%	0.052%	0.028%	
'CASSTGTSGSEQY'	0.005%	0.007%	0.242%	N.D.	
'CASSVSSYNEQF'	N.D.	N.D.	N.D.	1.276%	
'CASSWTSLPDEQY'	0.030%	N.D.	0.342%	N.D.	
'CASSYGGGASYEQY'	1.598%	0.170%	0.295%	0.197%	Yes
'CASSYIGSSYNEQF'	N.D.	0.149%	0.007%	0.071%	

'CASSYLDSYGYT'	N.D.	0.201%	0.015%	0.082%	
'CASSYSEGPYGYT'	N.D.	0.109%	N.D.	0.060%	
'CASSYSTNTEAF'	0.469%	0.060%	0.017%	0.082%	Yes
'CASTMLAKNTGELF'	0.001%	0.266%	0.054%	0.033%	Yes
'CASTNDGTGNTYEQY'	0.456%	0.105%	0.384%	3.944%	Yes
'CATFKGDRKAF'	0.998%	0.060%	0.024%	0.006%	Yes
'CATRGGGGETQY'	0.027%	0.002%	0.201%	N.D.	
'CSAPSGINYEQY'	0.553%	0.007%	0.004%	0.011%	Yes

**XRL**	**First**	**Second**	**Third**	**Fourth**	**TCPI$_{20}$=27/65**
'CAISEGDSPYEQY'	0.054%	N.D.	N.D.	0.408%	
'CAISESTRNQPQH'	0.155%	N.D.	N.D.	0.729%	
'CAISMDEF'	0.034%	0.015%	0.021%	0.243%	Yes
'CASGDRGFYNEQF'	N.D.	N.D.	0.474%	0.016%	
'CASGLSEGGNQPQH'	0.857%	0.420%	0.197%	0.172%	Yes
'CASIELSYSNQPQH'	0.189%	0.110%	0.044%	0.141%	Yes
'CASKAGTSVYGEQF'	N.D.	0.226%	0.018%	0.008%	
'CASKKGQGGTEAF'	N.D.	0.017%	1.256%	0.251%	
'CASLDRGLQPQH'	0.013%	0.005%	0.801%	0.227%	Yes
'CASMRTSAPDTQY'	N.D.	0.391%	0.060%	0.024%	
'CASQSRGMTF'	N.D.	N.D.	N.D.	0.329%	
'CASRDAGDYWEQY'	0.398%	0.120%	0.133%	0.110%	Yes
'CASRPGTETYEQY'	1.363%	0.023%	0.008%	N.D.	
'CASRPSRGGGYGYT'	N.D.	1.660%	0.147%	0.047%	
'CASRRDYSYNEQF'	0.007%	0.013%	0.575%	0.055%	Yes
'CASRTGTSDHEQY'	0.297%	0.367%	0.370%	0.243%	Yes
'CASSAGRMGTDTQY'	N.D.	N.D.	2.199%	0.024%	
'CASSDPYRGEPQH'	0.020%	0.012%	0.707%	0.133%	Yes
'CASSFMDRNEKLF'	N.D.	1.175%	0.116%	0.031%	
'CASSIEQNTEAF'	0.533%	0.017%	N.D.	N.D.	
'CASSLAGVGQETQY'	0.526%	0.181%	0.174%	0.102%	Yes
'CASSLDDAETDTQY'	N.D.	4.840%	0.072%	0.055%	
'CASSLEQNTEAF'	0.634%	0.010%	N.D.	N.D.	
'CASSLGQGRDTQY'	3.069%	0.632%	0.763%	1.520%	Yes
'CASSLGVTSYSEQY'	0.459%	0.012%	0.003%	N.D.	
'CASSLRLTLLNEQF'	0.040%	0.024%	0.031%	0.415%	Yes
'CASSLSGHDYEQY'	N.D.	0.246%	0.014%	0.016%	
'CASSLTDTQY'	0.027%	0.017%	0.844%	0.204%	Yes
'CASSLTTRIDEQY'	1.025%	0.457%	0.441%	0.439%	Yes
'CASSLVPGYDQPQH'	0.243%	0.029%	0.028%	N.D.	
'CASSPGLAGYGYT'	N.D.	0.610%	0.093%	N.D.	
'CASSPGQGAHSYEQY'	N.D.	N.D.	1.471%	N.D.	
'CASSPGQGYYGYT'	N.D.	0.004%	0.811%	0.172%	
'CASSPNGGYGYT'	N.D.	0.294%	N.D.	N.D.	

'CASSPQGANGYT'	N.D.	0.619%	0.086%	N.D.	
'CASSPQGASGYT'	N.D.	0.350%	0.034%	N.D.	
'CASSPQKAAAYRPDSGANVLT'	N.D.	N.D.	0.942%	0.016%	
'CASSPRGSGSESANVLT'	0.013%	0.001%	N.D.	0.948%	
'CASSPSTGLYNEQF'	N.D.	N.D.	N.D.	0.235%	
'CASSQAGTGFVEQY'	0.432%	0.581%	0.300%	0.517%	Yes
'CASSQAGTGWDTEAF'	0.013%	8.523%	0.038%	0.047%	Yes
'CASSQDGYGANVLT'	0.040%	N.D.	N.D.	0.400%	
'CASSQDRSSTDTQY'	N.D.	N.D.	0.730%	N.D.	
'CASSQEEVPYNEQF'	N.D.	N.D.	9.734%	0.039%	
'CASSQGQVSNTEAF'	N.D.	N.D.	1.104%	N.D.	
'CASSQGTSSGDTQY'	N.D.	N.D.	0.716%	0.008%	
'CASSQPGRQAF'	N.D.	N.D.	0.688%	0.008%	
'CASSQYDNEQY'	0.148%	0.145%	0.266%	0.690%	Yes
'CASSSPGSPSYNEQF'	N.D.	0.231%	0.005%	N.D.	
'CASSSRLAGGSETQY'	0.013%	0.025%	0.093%	0.415%	Yes
'CASSSRLAGGTDTQY'	0.216%	0.130%	0.216%	3.072%	Yes
'CASSTRLAGGTDTQY'	0.020%	0.025%	0.092%	0.964%	Yes
'CASSVAWDGNQPQH'	0.358%	0.207%	0.121%	0.016%	Yes
'CASSWTGPYEQY'	N.D.	0.233%	0.018%	N.D.	
'CASSYNGAYNEQF'	0.189%	0.126%	0.122%	0.094%	Yes
'CASSYSEGVSPNTEAF'	N.D.	0.023%	1.756%	0.274%	
'CASSYSTGQGRSTDTQY'	0.715%	0.011%	0.008%	0.125%	Yes
'CASSYYDPSVNTGELF'	9.019%	0.090%	0.031%	0.016%	Yes
'CASSYYPGRGNEQF'	0.783%	0.306%	0.324%	0.329%	Yes
'CASTATGAADTQY'	0.108%	0.078%	0.073%	0.243%	Yes
'CATKAGTSVNEQF'	N.D.	0.500%	0.026%	N.D.	
'CATSEHPGGFSQY'	0.027%	0.008%	1.146%	0.118%	Yes
'CATSGLAGETQY'	N.D.	N.D.	0.627%	N.D.	
'CSASGTDYNEQF'	N.D.	0.005%	0.548%	0.016%	
'CVAKGPPNEQF'	0.600%	0.168%	0.243%	0.204%	Yes
**YYS**	**First**	**Second**	**Third**		**TCPI$_{20}$=27/27**
'CAISESTRNQPQH'	0.245%	0.375%	0.077%		Yes
'CASGPGGSGANVLT'	0.677%	0.135%	0.618%		Yes
'CASLGTGTSGSYEQY'	1.512%	0.595%	0.206%		Yes
'CASRGDRGMDGYT'	0.797%	0.491%	0.472%		Yes
'CASRLAGGPGDTQY'	7.184%	2.710%	8.779%		Yes
'CASRTGGAGEAF'	13.502%	8.437%	18.227%		Yes
'CASRTRGAGEAF'	1.532%	0.863%	2.019%		Yes
'CASSGGSSAAGEQF'	0.495%	0.275%	0.756%		Yes
'CASSGRTSGSSYEQY'	0.392%	0.550%	0.112%		Yes
'CASSKTSGSGDEQF'	0.966%	1.150%	1.168%		Yes
'CASSLAGSGANVLT'	1.431%	1.431%	1.752%		Yes

'CASSLLVSGTYEQY'	0.192%	0.485%	0.026%	Yes
'CASSLPGVFGETQY'	3.387%	2.950%	5.463%	Yes
'CASSLTGDLYEQY'	0.535%	0.965%	1.280%	Yes
'CASSLYLPTDTQY'	0.001%	0.035%	1.709%	Yes
'CASSPNRGPPNEKLF'	0.882%	1.050%	1.014%	Yes
'CASSPTGSAYPGSCF'	0.267%	0.267%	0.438%	Yes
'CASSPTGVGIPGELF'	3.709%	3.117%	9.560%	Yes
'CASSQNQGAGGNQPQH'	0.916%	1.142%	2.766%	Yes
'CASSSGTTYEQY'	1.922%	0.406%	0.558%	Yes
'CASSSLGGQETQY'	0.590%	0.246%	0.369%	Yes
'CASSSLTSGRQETQY'	0.669%	0.234%	0.163%	Yes
'CASTNVGTGSNEQF'	0.103%	0.200%	0.412%	Yes
'CASTTSGSQEQY'	0.793%	0.744%	0.120%	Yes
'CATSRGAGRDQETQY'	0.514%	1.345%	6.751%	Yes
'CATSSGPSNTEAF'	3.603%	1.210%	2.963%	Yes
'CVSMDGNTGELF'	2.522%	1.558%	3.058%	Yes
**ZHF**	**First**	**Second**	**Third**	**$TCPI_{20}$=19/50**
'CASEPRDRGWNEQF'	0.408%	0.013%	0.003%	Yes
'CASFGNTVLYGYT'	0.498%	N.D.	N.D.	
'CASGQPQH'	0.476%	0.316%	0.049%	Yes
'CASGTGGSGANVLT'	0.048%	0.337%	0.003%	Yes
'CASGTSSEQF'	N.D.	0.283%	N.D.	
'CASNAGVWDEQF'	0.339%	N.D.	N.D.	
'CASSAAGNTEAF'	0.339%	N.D.	N.D.	
'CASSAFFEDTEAF'	0.005%	1.210%	0.570%	Yes
'CASSDFIGHGYYEQY'	0.312%	N.D.	N.D.	
'CASSDNQETQY'	0.386%	N.D.	N.D.	
'CASSEGREPQH'	N.D.	0.197%	2.074%	
'CASSESTYRSYNEQF'	0.402%	0.002%	0.003%	Yes
'CASSFVRGDRDDTQY'	N.D.	0.373%	2.375%	
'CASSKLSGRGELF'	0.048%	0.528%	7.912%	Yes
'CASSLEGTATKNIQY'	N.D.	0.077%	1.377%	
'CASSLGQNQPQH'	N.D.	0.092%	2.922%	
'CASSLLAGENYNEQF'	N.D.	0.706%	N.D.	
'CASSLSIGCCGYT'	N.D.	0.362%	0.332%	
'CASSLTGYNEQF'	0.053%	0.266%	0.106%	Yes
'CASSPDRGYNEQF'	N.D.	0.427%	0.001%	
'CASSPGENEQF'	0.498%	0.082%	0.009%	Yes
'CASSPGLHNEQF'	N.D.	1.003%	N.D.	
'CASSPGTSGTGSYEQY'	0.005%	0.071%	0.834%	Yes
'CASSPLFLPSSYNEQF'	0.720%	1.051%	0.666%	Yes
'CASSPSRTGGENEQF'	0.423%	0.004%	N.D.	
'CASSQDAGGYTGELF'	0.328%	N.D.	N.D.	

'CASSQGQRFVTEAF'	N.D.	0.176%	2.816%	
'CASSQGRTGETEAF'	N.D.	0.153%	1.713%	
'CASSQTTGIQRAF'	N.D.	0.013%	1.046%	
'CASSSFTSGSPDTQY'	0.312%	N.D.	N.D.	
'CASSSGQALTEAF'	N.D.	0.147%	0.764%	
'CASSSGTAGQPQH'	0.386%	N.D.	N.D.	
'CASSSLDGDTTYEQY'	N.D.	0.278%	0.896%	
'CASSSQGNTEAF'	N.D.	0.023%	0.699%	
'CASSTGHPNTEAF'	N.D.	0.017%	0.711%	
'CASSTQTGGYGYT'	N.D.	0.054%	2.266%	
'CASSTSGTGLRGYT'	0.529%	N.D.	N.D.	
'CASSVGLETGELF'	0.042%	1.357%	0.007%	Yes
'CASSVGLYNEQF'	0.487%	2.881%	0.004%	Yes
'CASSYETGGNEQF'	0.148%	0.601%	0.624%	Yes
'CASSYGGLYNEQF'	0.413%	0.008%	0.003%	Yes
'CASSYGQGAGGYT'	0.630%	1.026%	0.335%	Yes
'CASSYLREYEQF'	0.053%	0.574%	0.734%	Yes
'CASSYPQGKLF'	0.386%	0.013%	0.001%	Yes
'CASSYSGAGGLQETQY'	0.318%	0.065%	0.001%	Yes
'CASTTLSYSNQPQH'	N.D.	0.442%	0.738%	
'CASYDGLTTEAF'	N.D.	N.D.	0.749%	
'CATSRLAGEEQF'	0.037%	2.691%	17.683%	Yes
'CAVRGSISTGELF'	N.D.	0.027%	0.965%	
'CSASPQSYEQY'	N.D.	0.036%	0.709%	
